# Contrasting Rootstock-Mediated Growth and Yield Responses in Salinized Pepper Plants (*Capsicum annuum* L.) Are Associated with Changes in the Hormonal Balance

**DOI:** 10.3390/ijms22073297

**Published:** 2021-03-24

**Authors:** Amparo Gálvez, Alfonso Albacete, Cristina Martínez-Andújar, Francisco M. del Amor, Josefa López-Marín

**Affiliations:** 1 Institute for Agri-Food Research and Technology of Murcia (IMIDA), Department of Plant Production and Agrotechnology, c/Mayor s/n, E-30150 Murcia, Spain; amparo.galvez@carm.es (A.G.); franciscom.delamor@carm.es (F.M.d.A.); josefa.lopez38@carm.es (J.L.-M.); 2 Centro de Edafología y Biología Aplicada del Segura (CEBAS-CSIC), Department of Plant Nutrition, Campus Universitario de Espinardo, E-30100 Murcia, Spain; cmandujar@cebas.csic.es

**Keywords:** salinity, rootstock, pepper, vegetative growth, reproductive growth, ion homeostasis, hormonal balance

## Abstract

Salinity provokes an imbalance of vegetative to generative growth, thus impairing crop productivity. Unlike breeding strategies, grafting is a direct and quick alternative to improve salinity tolerance in horticultural crops, through rebalancing plant development. Providing that hormones play a key role in plant growth and development and stress responses, we hypothesized that rootstock-mediated reallocation of vegetative growth and yield under salinity was associated with changes in the hormonal balance. To test this hypothesis, the hybrid pepper variety (*Capsicum annuum* L. “Gacela F1”) was either non-grafted or grafted onto three commercial rootstocks (Creonte, Atlante, and Terrano) and plants were grown in a greenhouse under control (0 mM NaCl) and moderate salinity (35 mM NaCl) conditions. Differential vegetative growth versus fruit yield responses were induced by rootstock and salinity. Atlante strongly increased shoot and root fresh weight with respect to the non-grafted Gacela plants associated with improved photosynthetic rate and K^+^ homeostasis under salinity. The invigorating effect of Atlante can be explained by an efficient balance between cytokinins (CKs) and abscisic acid (ABA). Creonte improved fruit yield and maintained the reproductive to vegetative ratio under salinity as a consequence of its capacity to induce biomass reallocation and to avoid Na^+^ accumulation in the shoot. The physiological responses associated with yield stability in Creonte were mediated by the inverse regulation of CKs and the ethylene precursor 1-aminocyclopropane-1-carboxylic acid. Finally, Terrano limited the accumulation of gibberellins in the shoot thus reducing plant height. Despite scion compactness induced by Terrano, both vegetative and reproductive biomass were maintained under salinity through ABA-mediated control of water relations and K^+^ homeostasis. Our data demonstrate that the contrasting developmental and physiological responses induced by the rootstock genotype in salinized pepper plants were critically mediated by hormones. This will be particularly important for rootstock breeding programs to improve salinity tolerance by focusing on hormonal traits.

## 1. Introduction

To ensure food production for a continuously growing world population, crop production has been expanded to areas with low quality of soil and water, mainly caused by natural salinization [1]. Additionally, many traditional croplands are being increasingly affected by salinization due to the use of irrigation water with high concentrations of salts and also because of the effects of climate change, especially in the Mediterranean basin [2]. Salinity is one of the most important abiotic stresses, negatively affecting crop growth and productivity [2,3]. Indeed, growth rate reduction under salinity has been associated with stomatal limitation of photosynthesis provoked by the osmotic effect [4,5,6], while yield penalty has been associated with hormonal and metabolic factors [7,8].

Salinity tolerance strategies have been traditionally focused on selecting for above-ground traits, and only recently has the scientific community recognized the importance of modulating the root system to improve growth and productivity in horticultural crops, an approach that Gewin [9] named “underground revolution”. The direct manipulation of root traits can be done by grafting, a technique used for centuries in woody plants and in vegetable crops it has begun to be implemented as a common agricultural practice in the 20th century [10,11,12]. Several studies have demonstrated that grafting vegetable crops onto tolerant rootstocks can improve global plant performance and final productivity under different abiotic stress conditions [10,11,13,14,15,16]. Particularly, improved growth and yield stability responses mediated by the rootstock under salinity conditions have been described in different vegetable crops, such as tomato [17,18,19,20], pepper [21,22,23,24], cucumber [25,26], watermelon [27], and others [28]. These rootstock-mediated responses have been associated with a better status of the photosynthetic apparatus and increased sink activity [8,22], regulation of ion homeostasis [17,20,21,26], and modulation of hormonal balance [14,17,18,25,29].

Different hormone classes have been demonstrated to be modulated by grafting, thus improving salinity tolerance. Grafting a commercial tomato variety onto recombinant inbred lines derived from the wild-relative species *Solanum cheesmaniae* has shown to increase active cytokinins (CKs), such as *trans*-zeatin (tZ), and to reduce the ratio between the ethylene precursor 1-aminocyclopropane-1-carboxylic acid and abscisic acid (ACC/ABA) in the xylem, associated with delayed leaf senescence and improved growth and productivity under moderate salinity [17]. Importantly, those hormonal factors were closely associated with long-distance ion signaling of salinized grafted plants, since xylem K^+^ and the K^+^/Na^+^ ratio were positively loaded into the first principal component of statistical variability determining both leaf growth and photosystem II efficiency. Direct pieces of evidence of the role of root-derived CKs on growth and yield improvement were obtained with functional studies using transgenic rootstocks overexpressing the *IPT* gene, which encodes for a key enzyme of the CK biosynthesis, isopentenyl transferase. Besides, increasing the supply of ABA from the root through grafting wild-type scions onto transgenic tomato rootstocks with the *NCED* gene, encoding the 9-*cis*-epoxycarotenoid dioxygenase enzyme of the ABA biosynthesis [30], resulted in improved fruit yield under salinity conditions with respect to the self-grafted wild-type plants [14]. Using mutants impaired in jasmonic acid (JA) synthesis and signaling has shown that this hormone interacts with ABA and may also act as a root-to-shoot signal under drought and salinity stress [31]. Furthermore, using a tomato mutant (*res:* restored cell structure by salinity), which accumulates JA specifically in the root but not in the leaf, reciprocally grafted with its wild-type, has demonstrated that the growth and cellular alterations observed were root-based [32]. Importantly, the extreme tolerance to salinity observed in the *res* mutant correlated with increased mobilization of K^+^ from the root to the shoot and improved ion regulation mediated by JA, has also been supported by using transgenic rice lines with transcriptional regulation of JA signaling [33]. Other hormones have also been shown to regulate plant growth and productivity through long-distance signals from the root, such as salicylic acid (SA) and gibberellins (GAs), but their role in stress regulation is still scarce [34,35].

Although grafting has been used during the last few years as an efficient technique to improve vegetative growth and/or generative/reproductive rates of horticultural crops under salinity stress, no attempts have been made so far to determine the physiological mechanisms that determine the specific rootstock-mediated contribution to these productivity parameters. Previously, we have shown that fruit yield and quality of sweet pepper plants can be improved under water stress by selecting vegetative/generative/dwarfing rootstocks [36]. Therefore, we hypothesized that the regulation of vegetative-to-reproductive equilibrium can be optimized by modulating the hormonal balance through grafting, thus improving crop productivity under salinity stress. To test this hypothesis, we studied contrasting rootstock-mediated growth and yield responses of pepper plants subjected to moderate salinity stress associated with changes in the hormonal balance.

## 2. Results

### 2.1. Vegetative Growth-Related Parameters

**Figure 1:**
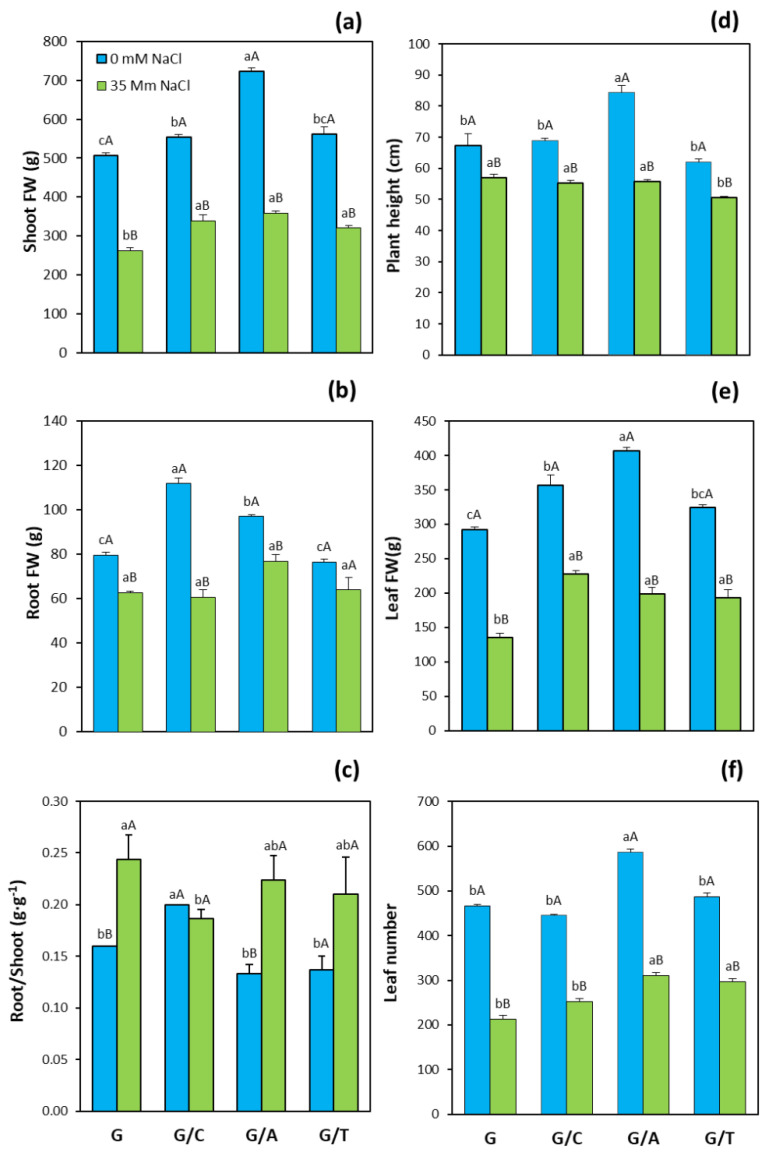
(**a**) Shoot fresh weight (FW), (**b**) root FW, (**c**) root-to-shoot ratio, (**d**) plant height, (**e**) leaf FW, and (**f**) leaf number of pepper plants of the commercial variety “Gacela F1” either non-grafted (G) or grafted onto commercial rootstocks Creonte (G/C), Atlante (G/A), and Terrano (T/A), and cultivated under control (0 mM NaCl) and salinity (35 mM NaCl) conditions. Bars show the means of five plants ± standard error. Different capital letters indicate significant differences due to the salt treatment while different small letters show significant differences among graft combinations according to Tukey’s test (*p* ≤ 0.05).

The use of different commercial rootstocks induced contrasting vegetative growth responses in sweet pepper plants. Plants grafted onto Atlante, Creonte, and Terrano significantly increased shoot fresh weight (FW) with respect to non-grafted plants under control conditions, with Atlante presenting the highest shoot FW among all grafting combinations (it being 40% higher than Gacela plants, Figure 1a). Similarly, although salinity provoked a significant reduction of shoot FW, the three commercial rootstocks had significantly higher shoot FW than non-grafted plants, with Atlante also showing the highest shoot biomass. Regarding root FW, only Creonte (by 43%) and Atlante (by 25%) presented significantly higher root biomass than non-grafted plants under control conditions (Figure 1b), while no significant differences were observed among graft combinations under salinity conditions. Vegetative biomass allocation, measured as the root-to-shoot ratio, was significantly higher in plants grafted onto Creonte under non-stress conditions (Figure 1c). Importantly, root to shoot ratio increased with salinity in all genotypes except for Creonte plants, which, in turn, presented the lowest (significant) ratio under stress conditions (Figure 1c).

Other growth and morphological parameters were also affected by the rootstock. For example, plant height was significantly superior than Gacela, only in plants grafted onto Atlante under control conditions (by 25%), whereas Terrano significantly decreased plant height (by 20%) under salinity (Figure 1d). As for the other shoot biomass-related parameters, leaf FW was significantly higher under control conditions in plants grafted onto Atlante, Creonte, and Terrano, from which Atlante induced the highest leaf biomass (by 35% compared to non-grafted plants, Figure 1e). Salinity provoked a significant decrease in leaf FW, but grafted plants on the three commercial rootstocks maintained significantly higher leaf FW (between 40% for Atlante and Terrano, and 65% for Creonte). Finally, leaf number, which indicates plant growth rate, was significantly higher than that of non-grafted plants under control conditions only in the Atlante graft combination (by 25%), while Terrano did not modify the leaf number and Creonte even decreased this parameter (Figure 1f). As for other growth-related parameters, salinity decreased leaf number but, considering the rootstock effect, it was significantly higher in plants grafted under Atlante and Terrano.

### 2.2. Evolution of Leaf Gas-Exchange Parameters

**Figure 2:**
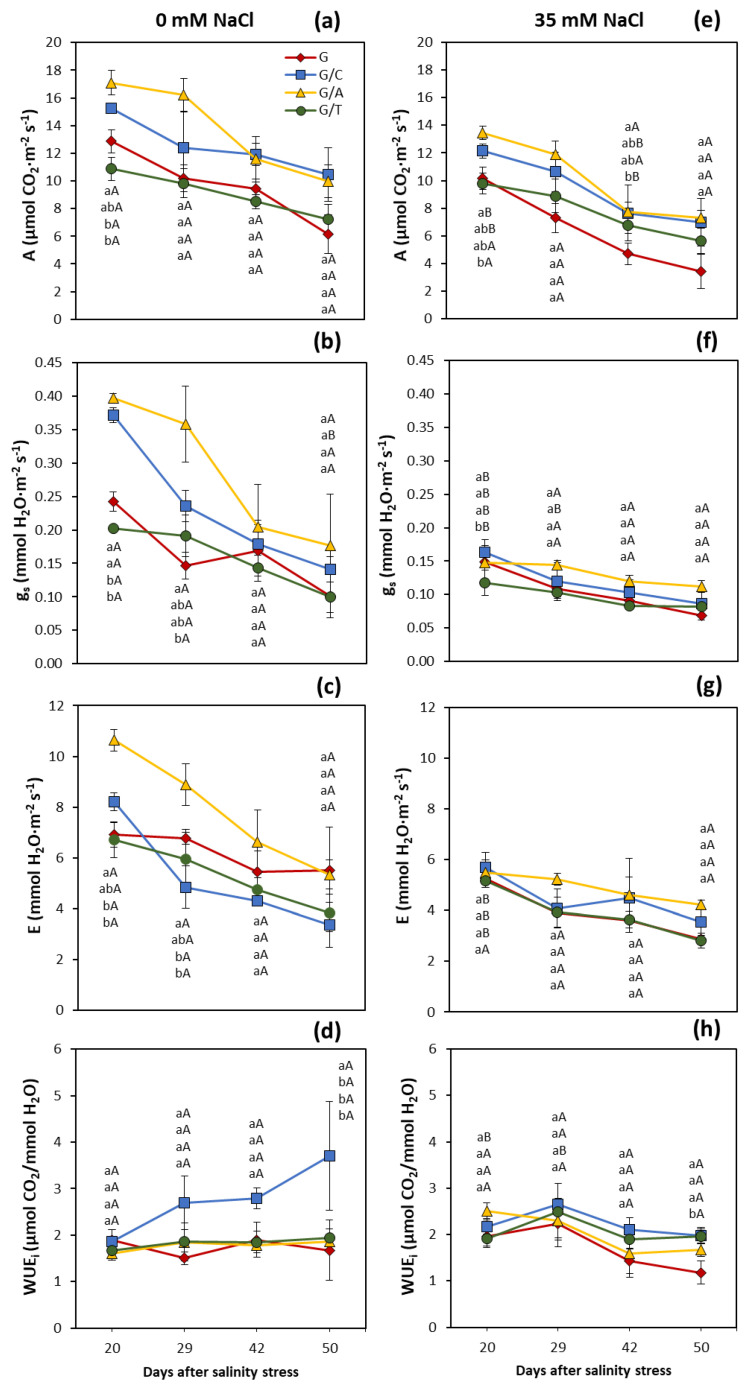
Evolution of (**a**,**e**) photosynthetic rate (*A*), (**b**,**f**) transpiration rate (*E*), (**c**,**g**) stomatal conductance (*g_s_*), and (**d**,**h**) intrinsic water use efficiency (WUE_i_) in leaves of pepper plants of the commercial variety “Gacela F1” either non-grafted (G) or grafted onto the commercial rootstocks, Creonte (G/C), Atlante (G/A), and Terrano (T/A), and cultivated under control (0 mM NaCl) and salinity (35 mM NaCl) conditions. Line points show the means of five plants ± standard error. Different capital letters indicate significant differences due to the salt treatment while different small letters show significant differences among graft combinations for each time-point according to Tukey’s test (*p* ≤ 0.05).

Grafting also produced differential leaf gas-exchange responses. Under control conditions, the photosynthetic rate decreased over time in all graft combinations and in non-grafted plants, but it was maintained at higher levels in plants grafted onto Atlante and Creonte throughout the whole period (Figure 2a). The photosynthetic rate also decreased over time under salinity conditions, with lower absolute values than those under control conditions, but this reduction was only significant in plants grafted onto Atlante and Creonte at the first analytical time-point (Figure 2e). Interestingly, non-grafted plants presented the lowest values of photosynthesis along the considered period, showing significant differences with grafted plants on the commercial rootstocks after 42 days of the onset of salinity stress (Figure 2e).

Stomatal conductance decreased over time, and this reduction was especially apparent in plants grafted onto Atlante and Creonte under control conditions. Indeed, after 20 and 29 days of salinity, those two graft combinations presented significantly higher stomatal conductance than self-grafted Gacela plants, whereas these differences vanished at the end of the analytical period (Figure 2c). Salinity strongly decreased stomatal conductance during the first 20 days of stress, while stabilization occurred from day 29 onwards. Importantly, although no differences were observed among graft combinations, plants grafted onto Atlante and Creoted maintained higher stomatal conductance over the period (Figure 2f).

As for the other gas-exchange parameters, the transpiration rate strongly decreased over time under non-stressed conditions (Figure 2c). Curiously, the transpiration rate dropped in plants grafted onto Creonte from day 29 onwards, whereas Atlante and non-grafted plants presented the highest values of transpiration at the last time-point of the considered period. Transpiration rate reduction over time was less apparent under salinity conditions, and plants grafted onto Atlante and Creonte retained transpiration with respect to those grafted onto Terrano and non-grafted Gacela plants (Figure 2g).

Intrinsic water use efficiency, calculated as the ratio between CO_2_ assimilation and stomatal conductance, increased during the considered period only in plants grafted onto Creonte, which, indeed, also presented the highest values under non-stressed conditions (Figure 2d). Importantly, all commercial rootstocks evaluated led to increased water use efficiency under salinity conditions, especially at the end of the period, despite the differences not being significant with respect to non-grafted plants (Figure 2h).

### 2.3. Fruit Yield-Related Parameters

**Figure 3:**
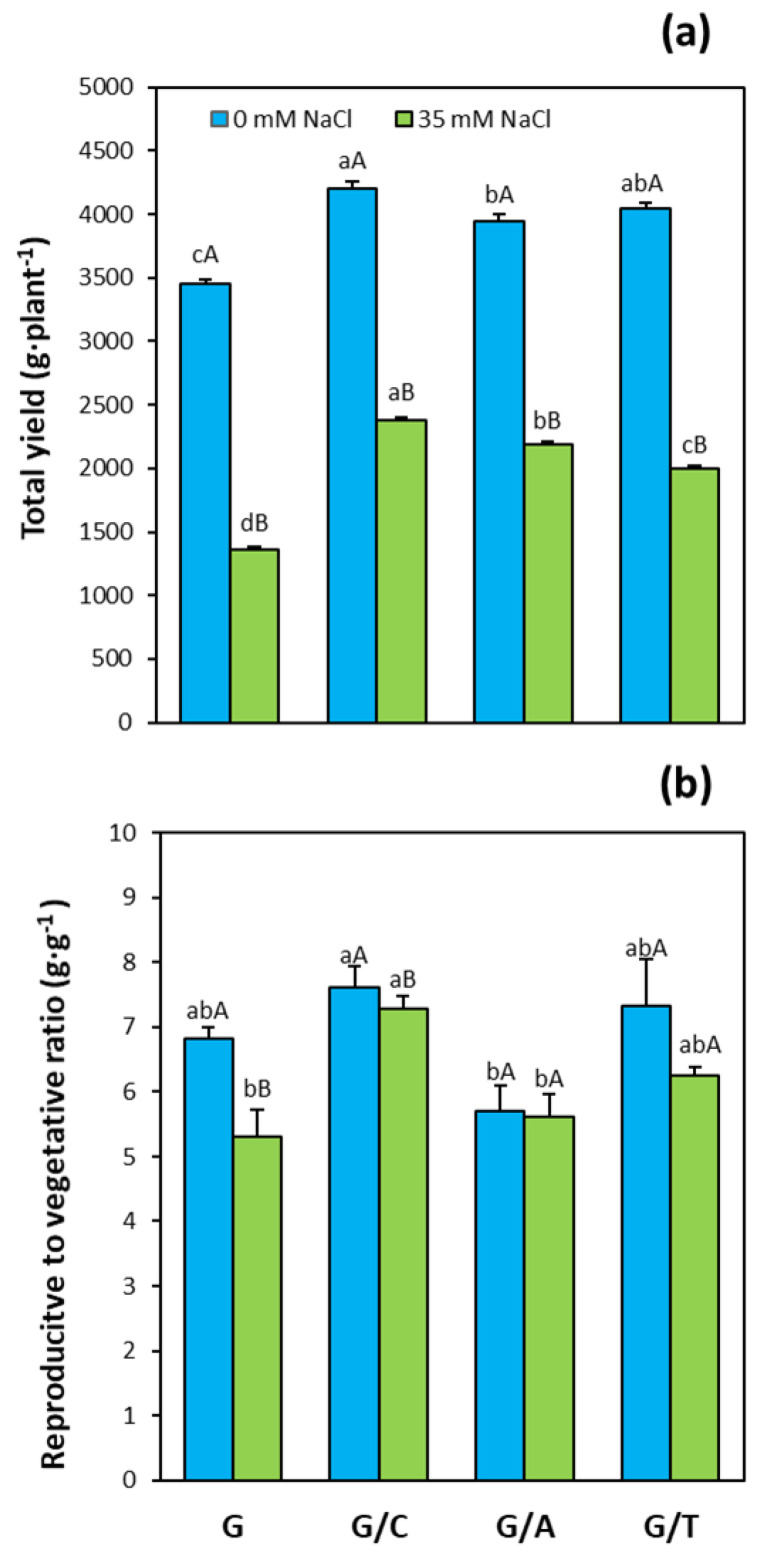
(**a**) Total yield, and (**b**) reproductive to vegetative ratio of pepper plants of the commercial variety “Gacela F1” either non-grafted (G) or grafted onto the commercial rootstocks, Creonte (G/C), Atlante (G/A), and Terrano (T/A), and cultivated under control (0 mM NaCl) and salinity (35 mM NaCl) conditions. Bars show the means of five plants ± standard error. Different capital letters indicate significant differences due to the salt treatment while different small letters show significant differences among graft combinations according to Tukey’s test (*p* ≤ 0.05).

Total yield, measured as the total weight of the fruits harvested per plant, was significantly higher in all graft combinations compared to non-grafted plants under control conditions, especially in plants grafted onto Creonte (by 30% higher, Figure 3a). Salinity treatment significantly decreased fruit yield in all graft combinations and non-grafted plants. However, plants grafted onto Creonte and Atlante presented significantly higher yield (by 60% and 75%, respectively) than non-grafted plants under salinity (Figure 3a).

An important derived parameter, the reproductive to vegetative ratio, which indicates the prevailing developmental process in the plant, was determined as the ratio between total vegetative biomass and the fruit yield. Under control conditions, plants grafted under Creonte showed the highest reproductive to vegetative ratio (being 18% higher than non-grafted plants), while plants grafted onto Atlante presented the lowest ratio (being 22% lower than non-grafted plants, Figure 3b). Salinity significantly reduced the reproductive to vegetative ratio, except for plants grafted onto Atlante. Notably, grafting plants onto Creonte resulted in a significantly higher reproductive to vegetative ratio than non-grafted Gacela plants (by 25%), whereas the other rootstock did not differentially affect this ratio under salinity (Figure 3b).

### 2.4. Mineral Nutrient Concentrations

Both grafting and salinity provoked changes in the concentrations of macro and micronutrients in the leaves. Under control conditions, plants grafted onto Atlante and Terrano presented higher NO^3−^ concentrations (by 40%) than non-grafted plants, whereas plants grafted onto Terrano showed higher PO_4_^3−^ (by 38%), and plants grafted onto Creonte and Atlante higher K^+^ concentrations (by 12%) than Gacela plants (Table 1). Mg^2+^ and SO_4_^2−^ also showed higher concentrations than non-grafted plants, while Ca^2+^ concentrations decreased in plants grafted onto commercial rootstocks. Notably, all previously described differences under non-stress conditions, although apparent, were not significant. Salinity provoked a general decrease in the leaf macronutrient concentrations, except for Mg^2+^. Interestingly, Atlante and Terrano significantly increased leaf NO_3_^−^ (by 40 and 14%, respectively) and K^+^ (by 28 and 30%, respectively) concentrations, whereas the other macronutrients were not significantly affected by grafting (Table 1).

None of the concentrations of the micronutrients analyzed were significantly modified by grafting under control conditions, although K^+^/Na^+^ ratio was significantly superior in plants grafted onto the commercial rootstocks than in non-grafted plants. The application of salinity significantly increased the concentrations of Cl**^−^**, Na^+^, and the K^+^/Na^+^ ratio in the leaves, while the other micronutrients, in general, were not affected by salinity (Table 1). Surprisingly, Fe^2+^ concentrations significantly increased with salinity in plants grafted onto Creonte and non-grafted plants, whereas leaves of plants grafted onto Terrano showed the lowest Fe^2+^ concentrations. Notably, Cl**^−^** and Na^+^ tended to be lower in all graft combinations with respect to non-grafted plants, while the K^+^/Na^+^ ratio significantly increased in plants grafted onto Creonte (Table 1).

### 2.5. Hormonal Profiling

**Figure 4:**
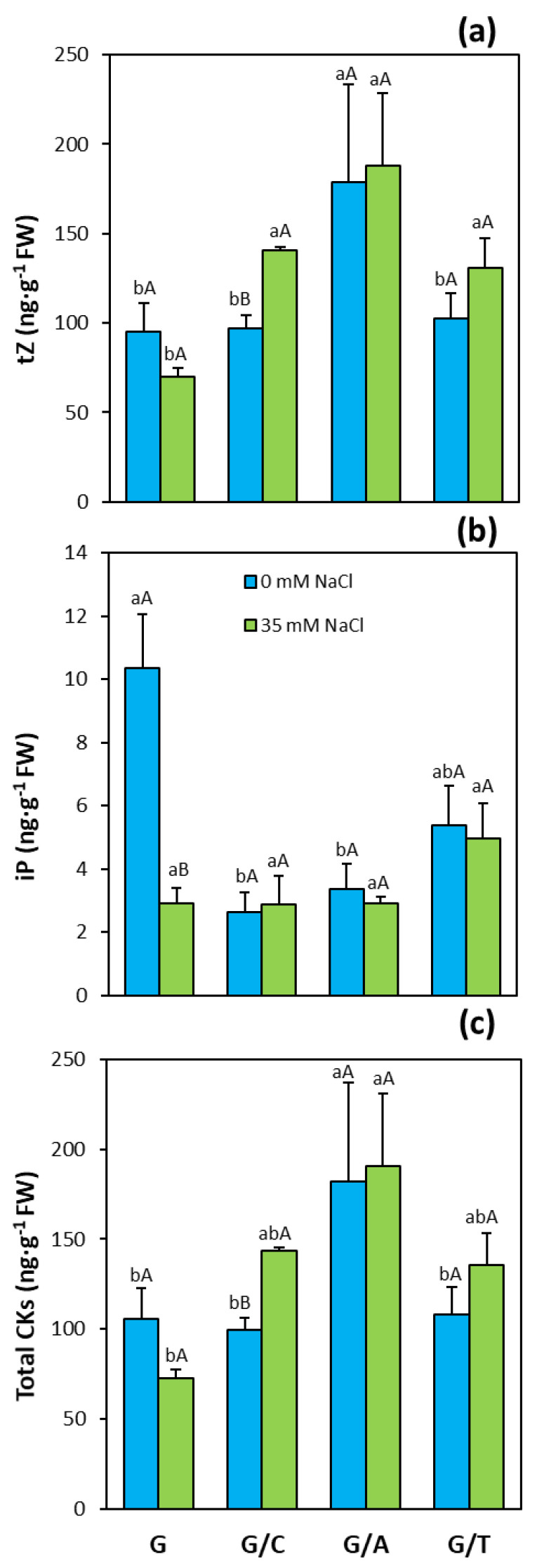
(**a**) *Trans*-zeatin (tZ), (**b**) isopentenyladenine (iP), and (**c**) total cytokinin (CKs) concentrations in leaves of pepper plants of the commercial variety “Gacela F1” either non-grafted (G) or grafted onto the commercial rootstocks Creonte (G/C), Atlante (G/A), and Terrano (T/A), and cultivated under control (0 mM NaCl) and salinity (35 mM NaCl) conditions. Bars show the means of five plants ± standard error. Different capital letters indicate significant differences due to the salt treatment while different small letters show significant differences among graft combinations according to Tukey’s test (*p* ≤ 0.05).

Three of the most active CKs in higher plants, tZ, RZ, and iP, were analyzed in leaves of pepper plants, but only tZ and iP concentrations were detected. The concentrations of tZ in non-stressed plants were significantly higher in Atlante than in the other graft combinations and non-grafted plants (Figure 4a). Under salinity conditions, plants grafted onto Atlante presented the highest tZ concentrations, whereas Creonte and Terrano also showed significantly higher tZ concentrations than non-grafted Gacela plants. The concentrations of the other active CK detected, iP, were significantly higher in non-grafted plants than in grafted plants onto the commercial rootstocks under control conditions (Figure 4b). However, salinity provoked a drop in the concentrations of iP in non-grafted plants, while no effect of salinity was observed in the grafted plants. Total CK concentrations, calculated as the sum of tZ and iP, were significantly higher in plants grafted onto Atlante than in the other graft combinations and the non-grafted plants under control conditions (Figure 4c). Similarly, under salinity, plants grafted onto Atlante showed the highest total CK concentrations (2.5-fold higher than non-grafted plants), followed by those of plants grafted onto Creonte and Atlante.

**Figure 5:**
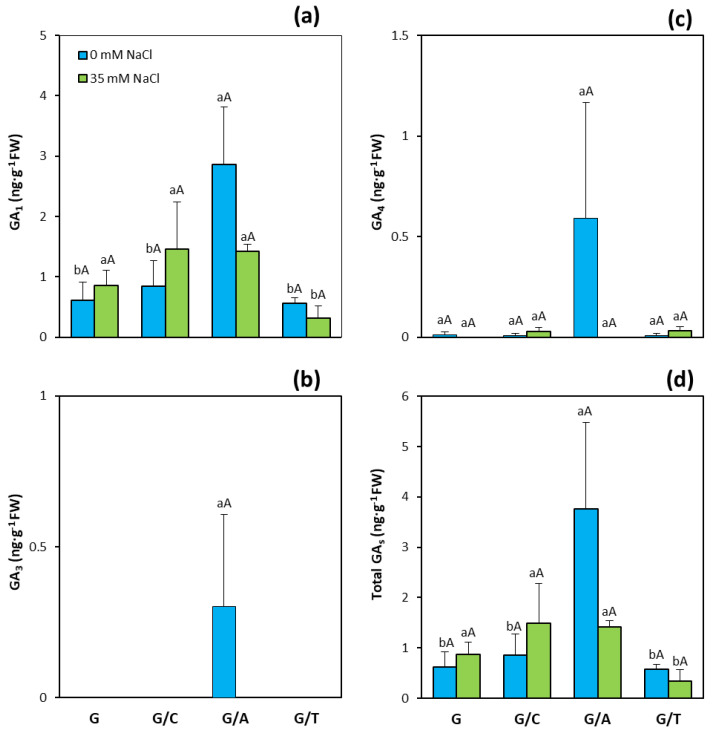
(**a**) Gibberellin A1 (GA_1_), (**b**) gibberellin A3 (GA_3_), (**c**) gibberellin A4 (GA_4_), and (**d**) total gibberellin (GAs) concentrations in leaves of pepper plants of the commercial variety “Gacela F1” either non-grafted (G) or grafted onto the commercial rootstocks Creonte (G/C), Atlante (G/A), and Terrano (T/A), and cultivated under control (0 mM NaCl) and salinity (35 mM NaCl) conditions. Bars show the means of five plants ± standard error. Different capital letters indicate significant differences due to the salt treatment while different small letters show significant differences among graft combinations according to Tukey’s test (*p* ≤ 0.05).

The balance of concentration of the three active GAs analyzed was also modified by grafting and salinity treatments. GA_1_ was the most abundant GA in the leaves of pepper plants. Plants grafted onto Atlante showed significantly higher GA_1_ concentrations than other graft combinations and non-grafted plants under control conditions (Figure 5a). Notably, under salinity stress, plants grafted onto Terrano presented the significantly lowest concentrations. GA_3_ was only detected in plants grafted onto Atlante under control conditions, whereas GA_4_ concentrations were very low except for plants grafted onto Atlance under control conditions (Figure 5b,c). Therefore, total GA concentrations, calculated as the sum of GA_1_, GA_3_, and GA_4_, were significantly higher in plants grafted onto Atlante under control conditions with respect to the non-grafted plants and the other graft combinations, while leaves of salinized plants presented the significantly lowest total GA concentrations when grafted onto Terrano (Figure 5d).

**Figure 6:**
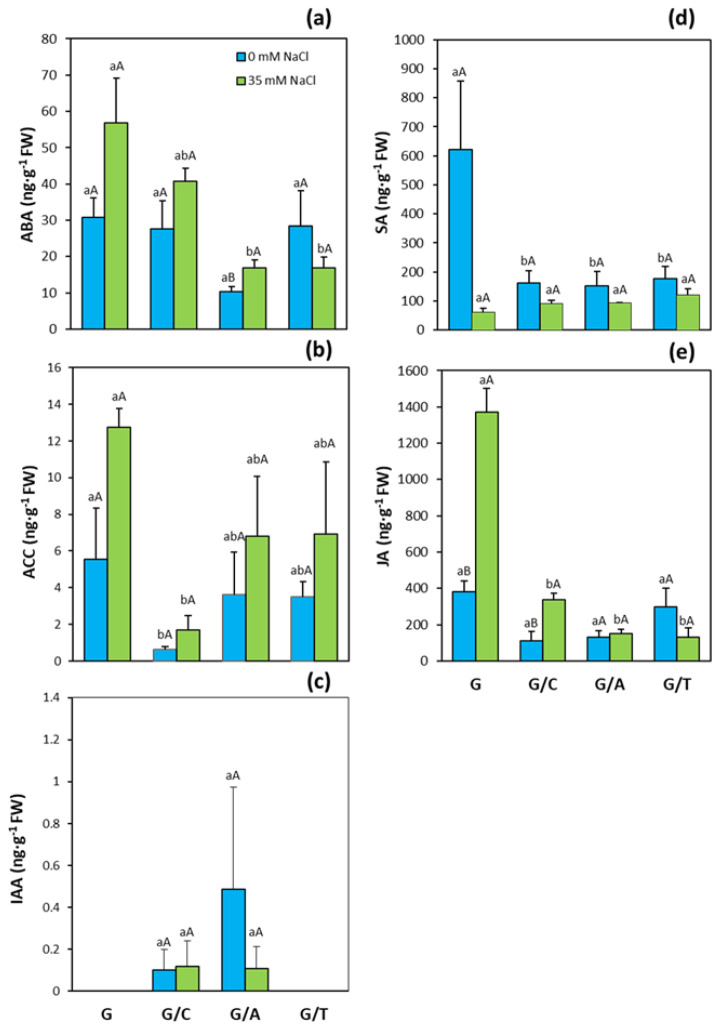
(**a**) Abscisic acid (ABA), (**b**) 1-aminocyclopropane-1-carboxylic acid (ACC), (**c**) indole acetic acid (IAA), (**d**) salicylic acid (SA), and (**e**) jasmonic acid (JA) concentrations in leaves of pepper plants of the commercial variety “Gacela F1” either non-grafted (G) or grafted onto the commercial rootstocks Creonte (G/C), Atlante (G/A), and Terrano (T/A), and cultivated under control (0 mM NaCl) and salinity (35 mM NaCl) conditions. Bars show the means of five plants ± standard error. Different capital letters indicate significant differences due to the salt treatment while different small letters show significant differences among graft combinations according to Tukey’s test (*p* ≤ 0.05).

ABA has been classically associated with stress responses under abiotic stress. Indeed, ABA concentrations increased with salinity in all genotypes analyzed except for plants grafted onto Terrano (Figure 6a). When plants were grown under control conditions, grafting plants onto Atlante significantly reduced ABA concentrations (Figure 6a). Under salinity conditions, non-grafted plants presented the highest ABA concentrations (3-fold higher than those of plants grafted onto Atlante or Terrano). The important role of ethylene as a stress-related hormone has been also extensively described. The concentrations of its direct precursor, ACC, have been directly correlated to those of ethylene [37]. ACC is a soluble molecule and thus can be measured with the same technology as the other hormone classes. Salinity increased ACC concentrations in all combinations, and plants grafted onto Creonte presented significantly lower ACC concentrations than non-grafted Gacela under both control and salinity conditions (Figure 6b). Regarding auxins, the most active one in higher plants, IAA, was only detected in plants grafted onto Creonte and Atlante rootstocks. Although no significant differences were observed between these two graft combinations, plants grafted onto Atlante under control conditions showed the highest IAA concentrations (Figure 6c).

SA and JA demonstrated to play important roles, especially in the defense against pests or pathogens. Under control conditions, SA concentrations decreased significantly in all graft combinations with respect to the non-grafted Gacela plants (Figure 6d). When plants were subjected to salinity, SA concentrations generally decreased, especially in Gacela (by 7.5-fold), but no significant differences among genotypes were observed (Figure 6d). In contrast, JA increased in non-grafted plants and plants grafted onto Creonte and Atlante when exposed to salinity conditions. This increase was especially apparent in non-grafted Gacela plants, whose JA concentrations in leaves were significantly higher (between 3.5- and 7.5-fold) than those of the plants grafted onto commercial rootstocks (Figure 6e).

### 2.6. Principal Component Analysis (PCA)

**Figure 7:**
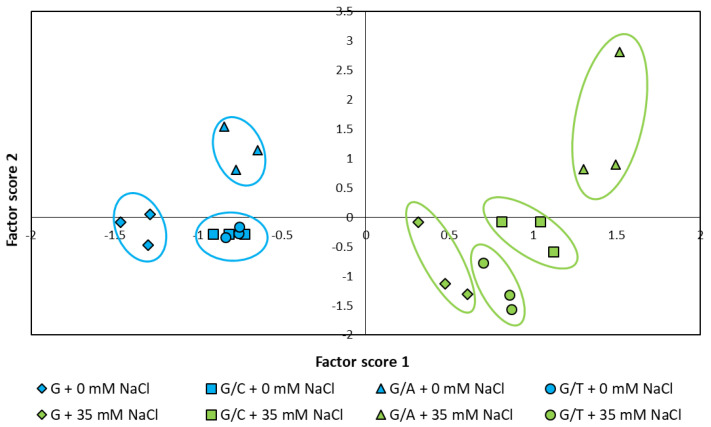
Bi-Plot representing the score values of pepper plants of the commercial variety “Gacela F1” either non-grafted (G) or grafted onto the commercial rootstocks Creonte (G/C), Atlante (G/A), and Terrano (T/A), and cultivated under control (0 mM NaCl) and salinity (35 mM NaCl) conditions.

To test statistically whether there existed differential patterns of the graft combinations assayed under control and salinity conditions, the set of data was subjected to a score PCA (Figure 7). This statistical test converts the normalized data into principal component scores through multiple dimension rotation that describes the major patterns of variation among graft combinations. The score PCA revealed a clear separation between the scores of the plants evaluated under control and salinity conditions and, in general, among those of the different graft combinations for each condition. The scores of plants grown under control conditions clearly separated non-grafted Gacela plants from plants grafted onto Atlante, while plants grated onto Creonte and Terrano clustered separately from the previous genotypes but not between each other (Figure 7). Importantly, salinity provoked a separation of the scores of plants grafted onto Creonte and Terrano in two different clusters, while maintaining the differential clustering of non-grafted plants and plants grafted onto Atlante.

**Figure 8:**
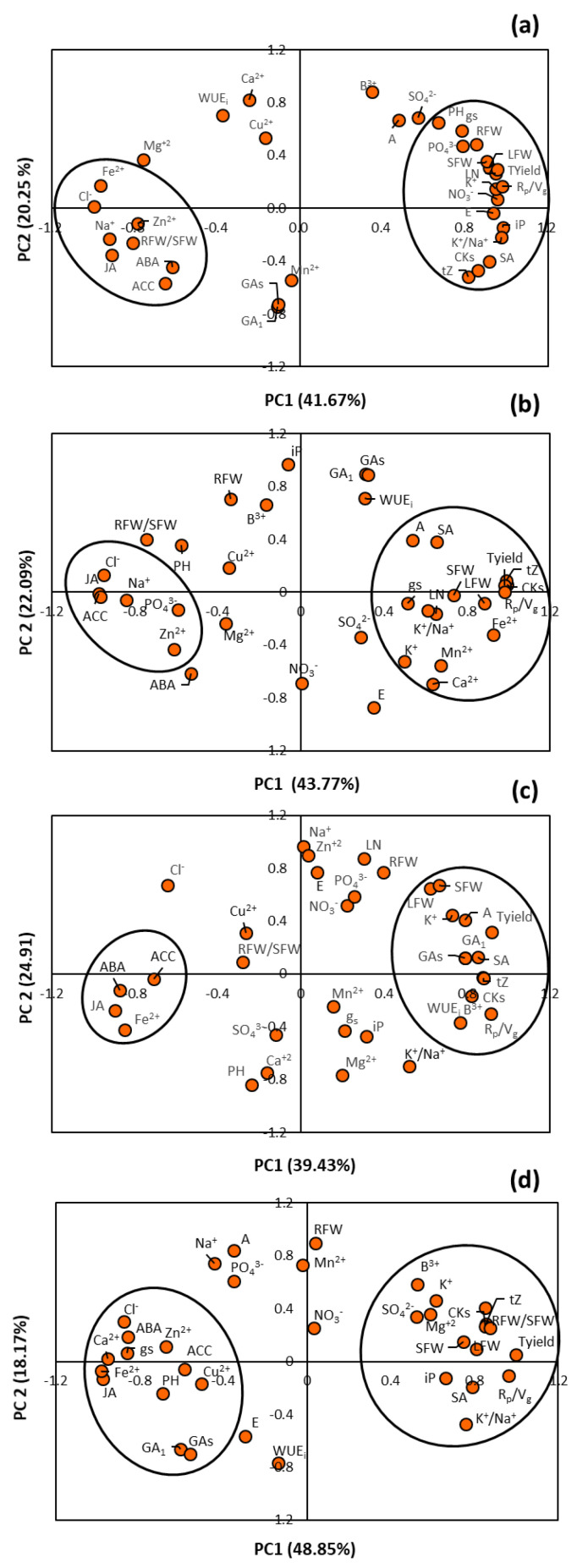
Two axes of a principal component (PC1, PC2) analysis showing the loadings of various growth-related, ionic, and hormonal variables (denoted by abbreviations) of pepper plants of the commercial variety “Gacela F1” either (**a**) non-grafted or grafted onto the commercial rootstocks (**b**) Creonte, (**c**) Atlante, and (**d**) Terrano, and cultivated under control (0 mM NaCl) and salinity (35 mM NaCl) conditions. SFW: Shoot fresh weight; RFW: Root fresh weight; LFW: Leaf fresh weight; PH: Plant height; LN: Leaf number; Tyield: Total yield; R_p_/V_g_: Reproductive to vegetative ratio; A: Photosynthetic rate; g_s_: Stomatal conductance; and E: Transpiration rate.

Furthermore, the dimensionality of the set of data was reduced by performing a principal component analysis (PCA) with the loadings of the variables used in this study (Figure 8). This mathematical algorithm allows identifying important ionic and hormonal traits regarding vegetative and reproductive rootstock-associated characteristics under salinity stress while maintaining the statistical variability [38]. Notably, the explained variability, which is the sum of that from the principal component (PC) 1 and PC2, ranged from 61.92% in non-grafted plants to 67.02% in plants grafted onto Terrano. In non-grafted Gacela plants, PCA revealed that growth- (Shoot FW, root FW, leaf FW, and leaf number) and yield-related parameters (total yield and reproductive to vegetative ratio) were closely associated with each other and with important ionic (NO_3_^−^, PO_4_^3−^, K^+^, and K^+^/Na^+^ ratio) and hormonal (SA, tZ, and total CKs) factors (Figure 8a). In contrast, the ionic (Na^+^ and Cl^−^) and hormonal (ABA, ACC, and JA) factors were inversely associated with the vegetative and reproductive traits of Gacela plants. The PCA of pepper plants grafted onto Creonte showed a divergence between some growth-related variables, especially root FW and root-to-shoot ratio, and the yield components (Figure 8b). Importantly, the active CK, tZ, and total CK content were the leaf traits most closely associated with the reproductive parameters, whereas Na^+^ and Cl^−^ (ionic), and JA and ACC (hormonal) traits varied in the opposite direction to that of the productivity parameters. As observed in plants grafted onto Creonte, grafting pepper plants with the commercial rootstock Atlante partially uncoupled reproductive parameters from the growth-related ones (Figure 8c). Notably, the photosynthetic rate, K^+^, and the hormonal traits (tZ, total CKs, GA_1_, total GAs, and SA) were associated with growth and yield, whereas the hormones ABA, JA, and ACC were inversely related. The loading PCA of pepper plants grafted onto Terrano revealed a close association of GAs with the height of the plants, which inversely varied with the shoot growth components (Figure 8d). Furthermore, both vegetative and generative biomass were associated with each other and with tZ and total CK content.

## 3. Discussion

Vegetable grafting has been demonstrated to influence growth and crop production as well as biotic and abiotic stress responses (revised in [12]). Modulating the vegetative to reproductive ratio through rootstocks could be an efficient strategy to cope with abiotic stress in horticultural crops. However, little is known about the physiological mechanisms implicated in the rootstock-mediated improvement of plant performance under stress regarding specific vegetative and/or reproductive components. Previously, we described rootstock-specific developmental changes in the scion variety “Herminio F1” of grafted pepper plants under water limitation and thermal stress conditions [36,39]. We found that the effects mediated by the commercial rootstock Creonte were related to the capacity of maintaining a high reproductive/vegetative ratio, while Atlante was a vigorous vegetative rootstock and Terrano a dwarfing-reproductive rootstock. In the present study, a different scion variety, “Gacela F1”, was grafted onto the same commercial rootstocks and assayed under salinity stress, while the rootstock effects on vegetative growth- and fruit yield-related parameters were similar to those encountered previously.

The improved vegetative biomass of pepper plants grafted onto the commercial rootstocks (Figure 1) can be explained by their higher photosynthetic rate, especially in plants grafted onto Atlante and Creonte under salinity stress (Figure 2a,e). Indeed, the photosynthetic rate of pepper plants grafted onto these two rootstocks clustered together in the PCA with the parameters controlling shoot growth (Figure 8b,c). We have previously shown that improved photosynthesis was associated with both the alleviation of growth inhibition under thermal stress in pepper plants grafted onto Creonte [39], and with the induction of vigor under drought in plants grafted onto Atlante [36]. Several studies have shown a rootstock-mediated effect on growth improvement of horticultural crops under abiotic stress through the control of photosynthesis. In this regard, salinity significantly and quickly decreased biomass and photosynthesis of pepper plants but to a lower extent in those grafted onto selected tolerant rootstocks [21,22,24]. Grafted pepper plants were able to maintain the shoot and root growth under salinity conditions by supporting the maintained photosynthetic performance [22]. Furthermore, screening several pepper genotypes as rootstocks revealed that water stress tolerance was mainly explained by net photosynthesis rate maintenance [40]. Similarly, the evaluation of contrasting physiological responses of sensitive versus drought- and salt-tolerant pepper accessions used as rootstocks demonstrated that osmotic and salt stress-tolerance responses were associated with a higher CO_2_ assimilation rate due to the rootstock-mediated protective capacity of the photosynthetic machinery [23,41,42].

Pepper plants grafted onto Creonte were the least affected by salinity in terms of shoot FW (Figure 1a) and total fruit yield (Figure 3a), giving rise to the maintenance of the reproductive to vegetative ratio (Figure 3b), and thus agronomical stability [8,14]. Importantly, these plants allocated vegetative biomass to the shoot under stress, as indicated by the root to shoot ratio (Figure 1c), suggesting that the control of source/sink relationships is an important physiological response of Creonte rootstock under salinity. Indeed, some works have demonstrated, by functional approaches, that the modulation of sink activity through sucrose-cleaving enzymes (invertases) improved vegetative growth and fruit yield in tomato plants growing under drought and salinity [7,43]. Improved growth and yield of plants overexpressing the cell wall invertase gene, *CIN1*, was explained by the metabolic control of photosynthesis, stomatal conductance, and water use efficiency, as seems to occur in pepper plants grafted onto Creonte (Figure 2).

Importantly, Terrano induced the compactness of the plant (Figure 1d), while maintaining shoot biomass (Figure 1a), fruit yield (Figure 3a), and thus reproductive to vegetative ratio (Figure 3b) under salinity. Dwarfing sweet cherry rootstocks have previously been shown to increase crop sustainability without affecting yield [44], whereas water relations have been demonstrated to play a key role in grain yield maintenance of a dwarfing sunflower cultivar grown under drought [45]. In the present study, the semi-dwarfing rootstock Terrano presented the lowest values of stomatal conductance and transpiration rate under salinity, thus improving water relations and water use efficiency (Figure 2d,h), and maintaining yield (Figure 3) [14]. In fact, the control of water relations and water use efficiency through grafting has been proposed as an efficient breeding strategy to improve drought and salinity stresses in both woody and horticultural crops [46,47].

The control of ion homeostasis, especially that of Na^+^ and K^+^, has been demonstrated to be crucial for salinity tolerance (recently reviewed by Hussain et al. [48]). In this regard, rootstock control of Na^+^ and K^+^ homeostasis has been shown to be key for improving growth and productivity in different vegetable crops [17,21,49]. In agreement with this, our study shows that, among all leaf macro and micronutrients analyzed, K^+^, Cl^−^, and Na^+^ were the most affected by both the salinity treatment and the rootstock genotype (Table 1). However, the PCA revealed a rootstock-mediated differential control of Na^+^ and K^+^ under salinity (Figure 8). In pepper plants grafted onto the generative rootstock Creonte, K^+^ and the K^+^/Na^+^ ratio clustered with the growth- and yield-related parameters, whereas Na^+^ and Cl^+^ grouped in an opposite cluster to that of the productivity parameters (Figure 8b). Notably, this rootstock reduced the accumulation of Na^+^ under salinity, suggesting an efficient mechanism of exclusion and/or reduced transport to the shoot of toxic ions (Table 1). Enhanced physiological responses and/or fruit yield under salinity mediated by rootstock were related to the re-establishment of ion homeostasis via Na^+^ exclusion in pepper [21,24], tomato [20,49], and cucumber [50]. This exclusion mechanism observed in plants grafted onto Creonte could be regulated by high-affinity potassium transporters (HKTs) that are crucial for the long-distance transport of Na^+^ in plants. Indeed, grafting a commercial cucumber variety onto rootstocks overexpressing the *CmHKT1;1* gene has been shown to limit Na^+^ transport from the rootstock to the scion and is proposed as a strategy for engineering salt tolerance in vegetable crops [26]. However, in both the vigorous Atlante and semi-dwarfing Terrano rootstocks, Na^+^ was uncoupled from the growth and yield responses, and K^+^ and the ratio K^+^/Na^+^ ratio took a leading role in terms of ionic homeostasis, as indicated by the PCA (Figure 8c,d). In grafted cucumber plants, the rootstock genotype has been shown to regulate the recirculation of K^+^, but not Na^+^ [51]. Albacete et al. [17] also demonstrated, by using a population of recombinant inbred lines, that rootstocks controlled K^+^ (but not Na^+^) homeostasis under moderate salinity in tomato. Indeed, the rootstock effects on leaf senescence and growth of tomato graft combinations occurred while Na^+^ concentration was still low in leaf xylem and at growth-compatible and non-severe toxic levels in the leaf tissue [17,52,53], suggesting that rootstock-mediated differences in hormonal factors were important in explaining the scion phenotype [17,38]. Importantly, the failure of the inflorescence to develop normally as well as other reproductive-related responses in grafted plants have been related to hormonal and metabolic factors rather than the accumulation of toxic ions, as seems to occur in this study with Atlante and Terrano [17,54].

Thus, root-derived hormonal traits can be exploited through biotechnological approaches and/or grafting to improve resource (water and nutrients) capture and plant development under resource-limited conditions [13,14]. Certainly, hormones play a key role in regulating salinity stress responses as rootstock-derived signals influencing scion growth and final crop productivity [29,55]. As stated before, our study revealed contrasting growth and yield responses to salinity in grafted pepper plants mediated by the rootstock. Besides the already discussed physiological and ionic responses, the modulation of the hormonal balance seems to be on the basis of such responses. Shoot growth allocation and improved yield responses under salinity provoked by the generative rootstock Creonte were associated with higher leaf concentrations of the active CK and tZ, and with total CKs (Figure 4a,c), and with a drop of the ethylene precursor ACC (Figure 6c) compared to the non-grafted Gacela plants. This has been further evidenced by the PCA, where CKs clustered with the productivity parameters, whereas ACC grouped in an opposite cluster (Figure 8b). Screening a recombinant population of rootstocks grafted onto a commercial tomato variety revealed an inverse regulatory mechanism of CKs and ACC in the control of leaf growth and senescence under moderate salinity [17,38]. Direct evidence of the role of root-derived CKs in improving shoot growth and fruit yield under salinity was functionally approached using transgenic tomato plants overexpressing the *IPT* gene that encodes a key enzyme of CK biosynthesis, through either transient *IPT* induction in the root or grafting [18]. Therefore, in pepper plants grafted onto Creonte, the modulation of CK and ACC (ethylene) levels seems to regulate salinity responses by a direct systemic effect on the overall plant status (biomass allocation) and by a local effect on fruit yield (sink activity), as previously reported by Albacete et al. [7,8].

The vigor responses observed in pepper plants grafted onto Atlante seem to be also modulated by CKs, in coordination with ABA. Indeed, plants grafted onto Atlante presented the highest concentrations of tZ and total CKs (Figure 4a,c) and clustered with the shoot biomass and yield parameters in the PCA (Figure 8c). Furthermore, these plants retained low leaf ABA concentrations (Figure 6a) that covaried inversely to CKs and the vegetative vigor and yield traits within the first variability component PC1 (Figure 8c). Notably, the significantly higher root biomass of Atlante under salinity (Figure 1b) would allow better access to water resources as it has been shown in open-field crops with modulated CK biosynthesis by transgenic rootstocks [29,56]. Furthermore, ABA sensitivity has been demonstrated to improve the water status in grafted tomato [14,57], pepper [24], and cucumber [25] plants under salinity. Thus, the coordinated modulation of ABA and CKs levels of pepper plants grafted onto Atlante would help to regulate stomatal aperture [58] and/or density [59], maintaining stomatal conductance (Figure 2f), transpiration rate (Figure 2g), and photosynthesis (Figure 2e) under salinity.

The coordinated modulation of ABA and CKs seems to be also on the basis of shoot biomass (Figure 1a) and fruit yield (Figure 3a) maintenance under salinity in plants grafted onto Terrano, despite reducing plant height (Figure 1d). Indeed, tZ and total CK concentrations significantly increased (Figure 4a) and clustered with the productivity parameters in the PCA (Figure 8d), while ABA significantly decreased (Figure 6a) and covaried inversely (Figure 8d). Importantly, the compaction effect provoked by Terrano in the scion variety seems to be mediated by GAs since GA_1_ and total GA concentrations were significantly reduced under salinity conditions (Figure 6a,d) and closely associated with the plant height in the PCA (Figure 8d). The GA control of plant growth under salinity has been demonstrated in tomato plants by the exogenous application of GA_3_ [60]. Besides, the regulation of the GA biosynthetic genes *Cla015407* and *JcGA20ox1* in grafted watermelon and *Jatropha curcas* plants, respectively, has been shown to enhance stem elongation accompanied by increased plant height [61]. In contrast, micrografting experiments between hypocotyls of Arabidopsis wild-type (Col-0) used as scions and GA-deficient mutant rootstocks have induced severe dwarfing and late flowering, suggesting a rootstock-mediated modulation of shoot GA endogenous concentrations [35]. Since GAs are negatively regulated by DELLA proteins [62], the ectopic expression of the gain-of-function mutant *Mhgai1* encoding a DELLA protein in tomato reduced plant height [63]. A grafting experiment demonstrated the long-distance movement of *Mhgai1* mRNAs and their effect on dwarfing of the scion genotype [64], as seems to occur in plants grafted onto Terrano. Therefore, developmental and yield responses induced by Terrano seem to be coordinately modulated by rebalancing CKs, ABA, and GAs under salinity.

## 4. Materials and Methods

### 4.1. Plant Material and Growth Conditions

Seedlings of sweet pepper (*Capsicum annuum* L.) from the commercial variety “Gacela F1” (Syngenta Seeds, Chicago, IL, USA) were grafted onto three commercial rootstocks, Atlante (Ramiro Arnedo, Calahorra, Spain), Terrano (Syngenta Seeds, USA), and Creonte (De Ruiter-Monsanto Seeds-Bayer Crop Science, Leverkusen, Germany), using the procedure of Japanese top graft procedure. Ungrafted Gacela plants were used as the control. Sixty days after sowing, grafted and ungrafted plants were transplanted to 20-L pots (50% sand, 30% peat, and 20% vermiculite) and transferred to an arch-shaped multi-span greenhouse covered with thermal polyethylene, located at the ‘Torreblanca’ experimental field in Torre Pacheco (Murcia, Spain, latitude: 37°45′ N; longitude: 0°59′ W). Plants were distributed in rows, with a separation of 40 cm between plants and 100 cm between rows. During the first two weeks after transplanting, plants were irrigated with a standard Hoagland nutrient solution, then half of the plants started to be irrigated with the nutrient solution supplemented with NaCl at a final concentration of 35 mM, while the other half continued to receive the original nutrient solution. We selected this salinity treatment on the basis of a previous experiment with different salinity levels, considering that pepper is a moderately sensitive crop to salinity [2,65]. Part of the solution (10–20%) freely drained depending on the solar radiation, to avoid the accumulation of salts in the substrate. Plants were grown up to the end crop cycle and three experimental blocks with 10 plants per treatment were evaluated. The air temperature and PAR radiation in each experimental unit were monitored during the growing cycle using a Testo 177-T4 temperature data logger (Testo SE & Co. KGaA, Lenzkirch, Germany) and a quantum sensor (LI-COR Inc., Lincoln, NE, USA), respectively.

### 4.2. Plant Growth and Fruit Yield Determinations

Plant growth-related parameters were recorded at the end of the experiment in 15 plants per treatment. Plant height was determined with a measuring tape and then root, shoot, and leaves within each plant were separated to determine their fresh weight (FW). Ripe fruits were harvested weekly during the last 5 weeks of the crop cycle, and the total fruit yield per plant was determined as the sum of the fruit yield for the 5 harvesting points.

### 4.3. Gas Exchange Measurements

Gas-exchange parameters were monitored at the generative stage in fully expanded leaves located just below the latest fruit that was set. Measurements were carried out at periodic time-points (4) after starting the salinity stress treatment. Net CO_2_ fixation rate (A_max_, µmol CO_2_·m^−2^·s^−1^), stomatal conductance to water vapor (g_s_, mmol H_2_O·m^−2^·s^−1^), and transpiration rate (E, mmol H_2_O·m^−2^·s^−1^) were measured in steady-state under conditions of saturating light (800 µmol·m^−2^·s^−1^) and 400 ppm CO_2_ with a LI-6400 instrument (LI-COR, Lincoln, NE, USA).

### 4.4. Ion Determination

Pepper leaf samples were oven-dried at 65 °C for 72 h and homogenized with a grinder. Anions were extracted from ground material (100 mg) with 20 mL of deionized water. Samples were analyzed in an ion chromatograph (Metrohm 861 Advanced Compact IC, Metrohm, Herisau, Switszerland) using a Metrohm Metrosep A Supp7250/4.0 mm column. For cation extraction, 50 mg of leaf material were digested at 80 °C in a HNO_3_/H_2_O_2_ solution (5/3, *v*/*v*). Cations were dissolved in 0.1 M HCl, diluted with de-ionized water, and filtered. Samples were analyzed by inductively coupled plasma optical emission spectrometry (ICP-OES, Vista-MPX, Varian, Australia).

### 4.5. Hormone Extraction and Analysis

Cytokinins (*trans*-zeatin, tZ, zeatin riboside, ZR, and isopentenyl adenine, iP), gibberellins (GA_1_, GA_3_, and GA_4_), indole-3-acetic acid (IAA), abscisic acid (ABA), salicylic acid (SA), jasmonic acid (JA), and the ethylene precursor 1-aminocyclopropane-1-carboxylic acid (ACC) were analyzed according to Albacete et al. [28] with some modifications. Briefly, 50 mg of freeze-dried plant material were dropped in 0.5 mL of cold (−20 °C) extraction mixture of methanol/water (80/20, *v*/*v*). Then, 10 µL of internal standard mix, composed of deuterated hormones ([^2^H_5_]tZ, [^2^H_5_]tZR, [^2^H_6_]iP, [^2^H_2_]GA_1_, [^2^H_2_]GA_3_, [^2^H_2_]GA_4_, [^2^H_5_]IAA, [^2^H_6_]ABA, [^2^H_4_]SA, [^2^H_6_]JA, [^2^H_4_]ACC, Olchemim Ltd., Olomouc, Czech Republic) at a concentration of 1 µg·mL^−1^ each, was added to the extraction homogenate. Solids were separated by centrifugation (20,000× *g*, 15 min, 4 °C) and re-extracted for 30 min at 4 °C in an additional 0.5 mL of the same extraction solution. Pooled supernatants were passed through Sep-Pak Plus †C18 cartridges (SepPak Plus, Waters, Mildford, MA, USA) to remove interfering lipids and part of plant pigments, and evaporated at 40 °C under vacuum to near dryness. The residue was dissolved in 0.5 mL of methanol/water (20/80, *v*/*v*) solution using an ultrasonic bath. The dissolved samples were filtered through 13-mm diameter Millex filters with a 0.22-µm pore size nylon membrane (Merck-Millipore, Darmstadt, Germany).

Ten µL of filtrated extract were injected in a U-HPLC-MS system consisting of an Accela Series U-HPLC (ThermoFisher Scientific, Waltham, MA, USA) coupled to an Exactive mass spectrometer (ThermoFisher Scientific, Waltham, MA, USA) using a heated electrospray ionization (HESI) interface. Mass spectra were obtained using the Xcalibur software version 2.2 (ThermoFisher Scientific, Waltham, MA, USA). For the quantification of the plant hormones, calibration curves were constructed for each analyzed component (1, 10, 50, and 100 µg·L^−1^) and corrected for 10 µg·L^−1^ deuterated internal standards. Recovery percentages ranged between 92% and 95%.

### 4.6. Statistical Analysis

The data were tested first for homogeneity of variance and normality of distribution. Analysis of variance and principal component analysis (PCA) were performed using SPSS for Windows (Version 25.0, SPSS Inc., Chicago, IL, USA). Means of different graft combinations and salinity treatments were compared using Tukey’s test at 0.05 of confidence level. The Varimax rotation method was used for the loadings-PCA while score-PCA was graphically plotted as a Bi-Plot score.

## 5. Conclusions

Grafting has been demonstrated to be a direct and efficient alternative to breeding for salinity stress tolerance in horticultural crops, through balancing the vegetative to reproductive ratio. Here we tested three commercial pepper rootstocks with contrasting growth and yield characteristics. The invigorating effect of Atlante has been associated with the improvement of photosynthesis and K^+^ homeostasis, coordinately controlled by CKs and ABA. Creonte induced a high reproductive to vegetative ratio leading to yield stability under salinity that could be explained by higher photosynthesis and an efficient mechanism of Na^+^ exclusion and/or transport limitation, mediated by the inverse regulation of CKs and ethylene (ACC). Finally, grafting pepper plants onto Terrano reduced plant height by limiting leaf GA accumulation while maintaining shoot biomass and fruit yield, associated with ABA-mediated improvement of water relations and K^+^ homeostasis under salinity. This work shows for the first time that the modulation of vegetative and reproductive development under stress by grafting is regulated by changes in the hormonal balance, which, indeed, will be of special interest to improve salinity tolerance and maintain food production within the actual climate change scenario.

## Figures and Tables

**Table 1:** Macronutrient and micronutrient concentrations in leaves of pepper plants of the commercial variety “Gacela F1” either non-grafted (G) or grafted onto the commercial rootstocks Creonte (G/C), Atlante (G/A), and Terrano (T/A), and cultivated under control (0 mM NaCl) and salinity (35 mM NaCl) conditions.

**Grafting**	**Salt Treatment**	**Macronutrients (mg·g^−1^ DW)**							
		**NO_3_^−^**	**PO_4_^3−^**	**K^+^**		**Ca^2+^**		**Mg^2+^**	**SO_4_^2−^**
G	0 mM NaCl	9.455 aA	11.879 aA	42.371 aA		42.935 aA		14.523 aA	2.828 bA
G/C		11.093 aA	15.524 aA	46.875 aA		45.561 aA		13.096 aB	3.007 bA
G/A		12.393 aA	15.675 aA	46.332 aA		39.731 aA		14.940 aB	3.311 abA
G/T		13.557 aA	16.429 aA	42.711 aA		37.659 aA		15.652 aB	5.389 aA
G	35 mM NaCl	1.517 bB	5.044 aB	24.617 bB		42.849 abA		17.020 aA	1.835 aA
G/C		1.500 bA	4.466 aB	27.916 bB		45.388 aA		16.036 aA	2.018 aB
G/A		2.189 aB	5.294 aB	31.719 aB		39.818 abA		17.097 aA	1.610 aB
G/T		1.723 aB	4.816 aB	32.060 aA		34.998 bA		19.152 aA	2.377 aB
**Grafting**	**Salt Treatment**	**Micronutrients (mg·g^−1^ DW)**							
		**Fe^2+^**	**Zn^2+^**	**Cu^2+^**	**Mn^2+^**	**B^3+^**	**Cl^−^**	**Na^+^**	**K^+^/Na^+^**
G	0 mM NaCl	0.321 aB	0.016 aA	0.0019 aA	0.076 aA	0.111 aA	20.532 aB	0.363 aB	116.543 bA
G/C		0.354 aB	0.017 aB	0.0009 aA	0.065 aA	0.108 aA	9.061 aB	0.253 aB	185.276 aA
G/A		0.271 aA	0.014 abB	0.0012 aA	0.063 aB	0.108 aA	13.704 aB	0.233 aB	198.849 aA
G/T		0.272 aA	0.011 bB	0.0003 aA	0.053 aB	0.096 aA	13.518 aB	0.233 aA	183.309 aA
G	35 mM NaCl	0.439 bA	0.033 aA	0.0019 aA	0.079 aA	0.094 aA	58.330 aA	7.365 aA	3.341 bB
G/C		0.631 aA	0.022 aA	0.0013 aA	0.106 aA	0.091 aA	40.366 bA	2.437 bA	11.425 aB
G/A		0.328 bcA	0.038 aA	0.0013 aA	0.078 aA	0.109 aA	53.823 abA	6.897 aA	5.417 abB
G/T		0.307 cA	0.021 aA	0.0009 aA	0.082 aA	0.105 aA	46.998 abA	5.462 abA	5.861 abB

Data are the means of five plants. Different capital letters indicate significant differences due to the salt treatment while different small letters show significant differences among graft combinations according to Tukey’s test (*p* ≤ 0.05).

## Data Availability

Not applicable.
